# Computer-Assisted Optimization of the Acetabular Rotation in Periacetabular Osteotomy Using Patient's Anatomy-Specific Finite Element Analysis

**DOI:** 10.1155/2018/9730525

**Published:** 2018-02-04

**Authors:** Sung-Jae Park, Sung-Jae Lee, Wen-Ming Chen, Jung-Hong Park, Yong-Soo Cho, Taejin Shin, Soon-Yong Kwon

**Affiliations:** ^1^Central R&D Center, Corentec Co. Ltd., Banpo-dong, Seocho-gu, Seoul 06541, Republic of Korea; ^2^Department of Biomedical Engineering, Inje University, Obang-dong, Gimhae 50834, Republic of Korea; ^3^Department of Biomedical Engineering, University of Shanghai for Science and Technology, Shanghai 200093, China; ^4^R&D Institute, YM Yangsan Machinery Ltd., Jeonggwan-eup, Gijang-gun, Busan 46027, Republic of Korea; ^5^Department of Orthopaedic Surgery, St. Mary's Hospital, Catholic University, Yeouido-dong, Yeoungdeungpo-gu, Seoul 07345, Republic of Korea; ^6^Department of Orthopaedic Surgery, St. Paul's Hospital, Catholic University, Jeonnong-dong, Dongdaemun-gu, Seoul 02559, Republic of Korea

## Abstract

Periacetabular osteotomy (PAO) is a complex surgical procedure to restore acetabular coverage in the dysplastic hip, and the amount of acetabular rotation during PAO plays a key role. Using computational simulations, this study assessed the optimal direction and amount of the acetabular rotation in three dimensions for a patient undergoing PAO. Anatomy-specific finite element (FE) models of the hip were constructed based on clinical CT images. The calculated acetabular rotation during PAO were 9.7°, 18°, and 4.3° in sagittal, coronal, and transverse planes, respectively. Based on the actual acetabular rotations, twelve postoperative FE models were generated. An optimal position was found by gradually varying the amount of the acetabular rotations in each anatomical plane. The coronal plane was found to be the principal rotational plane, which showed the strongest effects on joint contact pressure compared to other planes. It is suggested that rotation in the coronal plane of the osteotomized acetabulum is one of the primary surgical parameters to achieve the optimal clinical outcome for a given patient.

## 1. Introduction

Developmental dysplasia of the hip (DDH) manifests various morphological abnormalities including acetabular dysplasia, decreased acetabular coverage of the femoral head, excessive femoral anteversion, increased neck-shaft angle, and shortened femoral neck [1]. Patients with DDH are usually adolescents or young adults with congenital deformities. When left untreated, DDH can cause secondary osteoarthritis due to prolonged exposure to increased contact stresses on the articular cartilage in the hip joint [2–4].

Periacetabular osteotomy (PAO) is one of the preferred joint-preserving techniques known to correct multiaxial hip deformities in DDH patients [5–7]. A PAO involves osteotomy at the periphery of the ilium and the ischium and followed by rotation of the acetabulum in three dimensions. Studies have shown that PAO could effectively reduce the joint load and relieve abductor muscle forces through the medial translation of the hip joint center [8].

To achieve the optimal surgical outcome, joint congruency between the femoral head and the acetabulum must be established. Normally, preoperative (Pre-OP) information including the location of the osteotomy and subsequent rotations of the acetabulum in terms of the direction and the amount have to be determined [9]. Clinical studies showed that individualized Pre-OP planning of PAO could improve surgical outcomes [10–17]. Unfortunately, quantitative information regarding the optimal rotational parameters remain unclear. As a result, surgical planning still largely relies on the experience and decision of the clinicians. Further, Pre-OP planning and postoperative (Post-OP) assessment usually depend on the radiographic X-ray imaging which are essential in two dimensions, as opposed to the three-dimensional orientation and acetabular rotations for hip realignment during the surgery.

Pre-OP planning was first introduced by Langlotz et al. [10], which generally involves measurement of morphological parameters such as center-edge (CE) angle in X-ray images [11–13]. In contrast, recent development of Pre-OP planning based on biomechanical modelling permits a more quantitative solution. Biomechanical information, such as tissue stresses, contact area, and contact pressure in the hip joint, can be predicted through computational simulations, such as finite element (FE) analysis [12, 15, 16]. Zou et al. [15] constructed hip FE models for five patients with DDH to investigate the optimal location of the acetabulum in PAO in relation to CE angle. Zhao et al. [16] investigated the effect of PAO on von Mises stresses on the cortical bone of the acetabulum. The above studies, however, only considered the two-dimensional acetabulum rotations.

In a recent study [17], we constructed an anatomy-specific FE model based on computed tomography (CT) images collected from a patient who underwent PAO surgery. In that study, we quantitatively determined the biomechanical parameters, including hip joint contact area, contact pressure, and peak von Mises stress, before and after the PAO surgery. However, our previous model used a simplified approach by limiting the acetabular rotation in a single anatomical plane, and the actual acetabular rotations during the surgery which are in three dimensions were not considered. Thus, the effects of acetabular rotation in different anatomical planes on joint contact mechanics remain unclear.

This study aims to investigate the principal axes of rotation of the acetabulum and to assess the optimal amount of the acetabular rotation in three dimensions in a dysplastic hip model. To this end, a range of rotation of the osteotomized acetabulum during PAO was calculated using the patient's anatomy-specific FE models [17]. A series of FE analyses were performed based on the measured anatomical angles and changes in the joint coverage areas, and contact stresses were evaluated due to incremental rotation of the osteotomized acetabulum in three dimensions.

## 2. Materials and Methods

Hip dysplasia is known to affect the structural geometry of the femoral head and the acetabulum. To capture the realistic geometry of a diseased hip, CT images were collected from a 42-year-old female patient (body weight of 52 kg) who was diagnosed with DDH and underwent PAO surgery at Fukuoka University Hospital (Fukuoka, Japan). The Pre-OP scan was performed for the hip and the pelvis of the patient using a clinical scanner (Aquilion 64, Toshiba Medical System Corp., Japan) at a resolution of 0.398 mm and a slice spacing of 2.0 mm. The Post-OP images were obtained 2 months after the surgery from the same patient using the same scanning parameters.

The focus in this paper is twofold: first, based on the Pre- and Post-OP CT images to calculate the amount of actual acetabular rotation (ACR) during the PAO and second, based on the actual ACR to guide the development of a series of Post-OP computational models of the dysplastic hip following various acetabular rotations in three dimensions. Using FE analysis, the biomechanical responses obtained from the Post-OP models including peak contact pressure and contact area were compared to the Pre-OP model to determine the efficacy of acetabular rotations along different axis during PAO.

### 2.1. Three-Dimensional Rotations of the Acetabulum due to PAO

**Figure 1:**
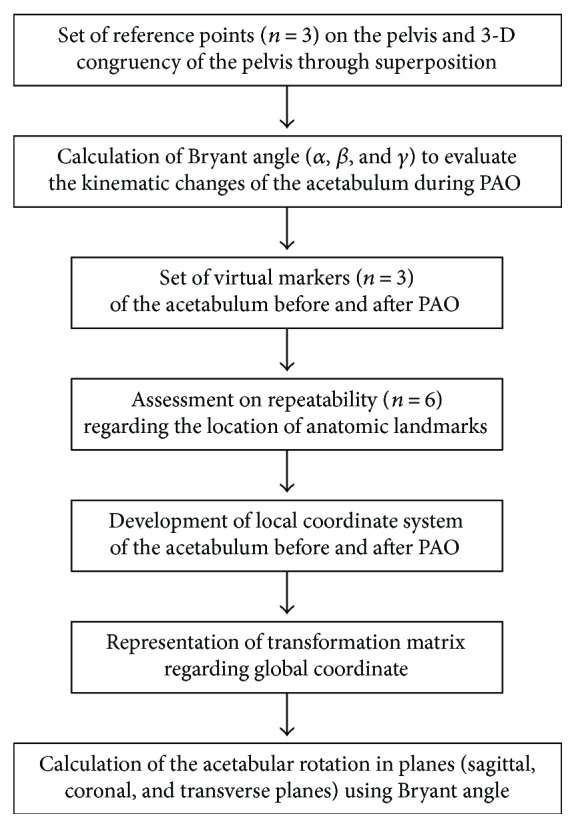
Flow chart for three-dimensional rotational calculation of the osteotomized acetabulum during PAO.

We implemented an image registration method for the calculation of three-dimensional rotations of the osteotomized acetabulum during PAO. The detailed procedures were performed as described in Figure 1.

While the Pre- and Post-OP CT images were collected at the same resolution, the scanning position was changed. To ensure congruency, the Pre- and Post-OP images were realigned such that the pelvis (excluding the acetabulum) before and after PAO was registered by superposition in the commercial image-processing software Mimics (Materialise, Louvain, Belgium). Using the built-in image registration function, the spatial position and orientation of the pelvis in Pre- and Post-OP CT images were realigned.

**Figure 2:**
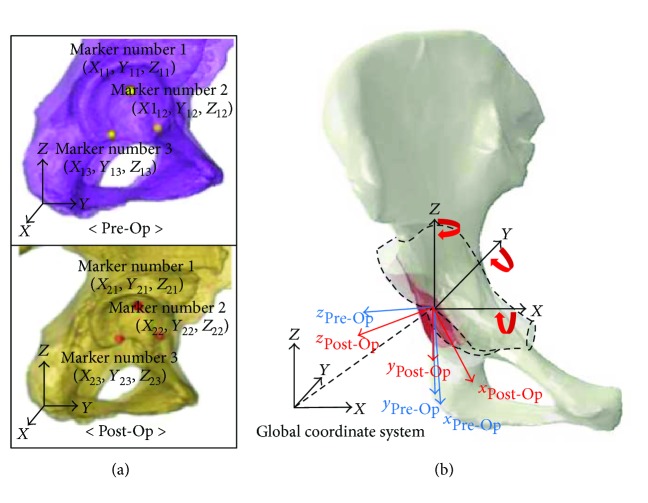
Reconstructed solid models of the pelvis based on the Pre- and Post-OP CT scans. The extent of osteotomy of the acetabulum was shown with a dotted line. (a) Locations of the anatomic landmarks in Pre- and Post-OP models in a lateral view (mark number 1 for acetabular fossa; mark number 2, and mark number 3 for the acetabular anterior and inferior sites, resp.); (b) the global coordinate system (X, Y, and Z; sagittal, coronal, and transverse planes) were shown. Three-dimensional rotations of the acetabulum were described by three Bryant angles along each axis.

To calculate the amount of acetabular rotations during PAO, two geometrical models, that is, solid models, were built based on reconstruction of the two sets of realigned Pre- and Post-OP images using a previously established protocol [17]. Virtual markers (*n* = 3) were set at the end of acetabular fossa (marker number 1), inferior (marker number 2), and anterior sites (marker number 3), which were clearly identifiable in both Pre- and Post-OP models (Figure 2). To increase the accuracy in placing these markers, three-dimensional geometrical objects, that is, spheres with radius of 3.0 mm, were used to locate the anatomic landmarks of the acetabulum. Three-dimensional coordinates at the center of sphere in the Pre- and Post-OP models were extracted to indicate the spatial location of these anatomic landmarks.

To evaluate the repeatability of individual marker placement, the interobserver variability was assessed in six independent observers. Repeatability between each landmark was evaluated [18] by repeatedly using coordinates of landmarks set (*X_ij_*, *Y_ij_*, and *Z_ij_*: *i* refers to before and after PAO, *i* = 1  and  2; *j* denotes to anatomic landmark, *j* = 1, 2, and  3) placed by the observers (${\dot{X}}_{ij}$, ${\dot{Y}}_{ij}$, and ${\dot{Z}}_{ij}$) using (1). This equation calculates the Euclidean distance between two landmarks in the three-dimensional space. The interclass correlation coefficient (ICC) on position of the virtual markers was also measured and assessed to confirm interobserver variations using statistical software (SPSS 22, SPSS Inc., USA).

(1) $$\begin{array}{c} Euclidean\;distances=\sqrt{{\left(X_{ij}-{\dot{X}}_{ij}\right)}^{2}+{\left(Y_{ij}-{\dot{Y}}_{ij}\right)}^{2}+{\left(Z_{ij}-{\dot{Z}}_{ij}\right)}^{2}}. \end{array}$$

Among the most common parameters used to describe the angular orientation of a body in three dimensions are Euler angles [19]. Using Euler angles, the angular orientation of a given body-fixed (i.e., local) coordinate system can be envisioned to be the result of three successive rotations. However, in the body-fixed coordinate system, the sequence of rotations used to define the final orientation of the coordinate system is to some extent arbitrary. For example, the Euler angles which act as a set of three independent body-fixed coordinates are altered as the initial body-fixed coordinate system changes during body's three-dimensional rotations.

Therefore, we calculated the angular orientation relative to the global coordinate system, which is defined as Bryant angles [19]. A local coordinate system was first defined for ease of description of the calculation. The vector connecting marker number 1 and marker number 2 defined the *x*-axis of local coordinate system. The vector connecting marker number 2 and marker number 3 determined vector **q**. Cross product of vectors **x** and **q** determined vector of *z*-axis by applying the right-handed rule. Likewise, the *y*-axis vector of local coordinate was determined by applying the cross products of vectors **x** and **z**, as shown in

(2) $$\begin{array}{c} \vec{x}\times \vec{q}=\vec{z},\\ \vec{x}\times \vec{z}=\vec{y}. \end{array}$$

By assuming rigid body motion, a transformation matrix **T** [20] for the local coordinate system in describing acetabulum rotation before and after surgery reads as follows:

(3) $$\begin{array}{c} \begin{array}{c} T=\left[\begin{array}{ccc} R_{11} & R_{12} & R_{13}\\ R_{21} & R_{22} & R_{23}\\ R_{31} & R_{32} & R_{33} \end{array}\right]=\left[\begin{array}{ccc} \cos\beta \cos\beta & \cos\alpha \sin\beta \sin\gamma -\sin\alpha \cos\gamma & \cos\alpha \sin\beta \cos\gamma -\sin\alpha \sin\gamma \\ \sin\alpha \cos\beta & \sin\alpha \sin\beta \sin\gamma -\cos\alpha \cos\gamma & -\sin\alpha \cos\beta \\ -\cos\alpha \sin\beta \cos\gamma -\sin\beta \sin\gamma & \cos\alpha \sin\beta \sin\gamma -\sin\alpha \cos\gamma & \cos\alpha \cos\beta \end{array}\right] \end{array}, \end{array}$$

where each column in matrix indicates the unit vector on *x*-, *y*-, and *z*-axis. And three-dimensional movement of the acetabulum was expressed in Bryant angle (*α*, *β*, and *γ*) and cosine, sine function with regard to global coordinate system [20]. The Bryant angles describes flexion (*x*-axis), adduction (*y*-axis), and external rotation (*z*-axis) of the hip movement. Thus, the relative acetabular rotation (*R*) could be obtained by multiplying the inverse transformation matrix **T**^−1^ as follows:

(4) Rpostpre=T−1×RpostG,

where *G* represents to global coordinate system; pre and post denote to before and after surgery, respectively. Based on matrix components (as in (3)), Bryant angle of the osteotomized acetabular rotation about three orthogonal planes was calculated using arctangent function as follows:

(5) $$\begin{array}{c} \alpha =\arctan\frac{R_{23}}{R_{33}},\\ \beta =\arctan\left({\left(\frac{R_{13}}{{R_{23}}^{2}}+{R_{23}}^{2}\right)}^{1/2}\right),\\ \gamma =\arctan\left(-\frac{R_{21}}{R_{11}}\right). \end{array}$$

### 2.2. Construction of Post-OP FE Models

**Figure 3:**
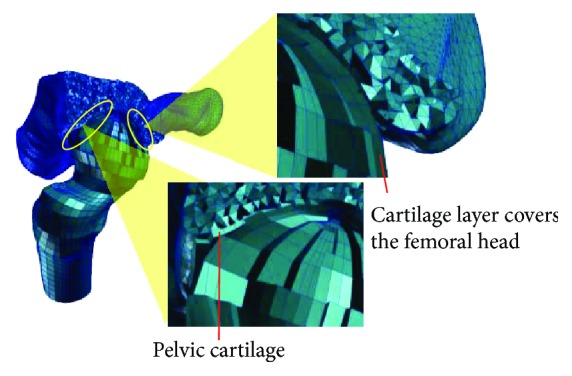
A cutaway view of the FE model illustrating interior mesh for the layers of articular cartilage covering the femoral head and acetabulum.

A previously constructed Pre-OP FE model was unitized to provide the baseline geometry of a dysplastic hip [17]. This Pre-OP model accurately captures the geometry of the diseased bone-cartilage interface. Bone tissues were differentiated from soft tissues in relation to the threshold in grey scale value which is equivalent of 226~3017 Hounsfield units (HU). Furthermore, subdivision between the cortical and cancellous bones of the proximal femur was made based on the threshold value for the cortical bone (662–1988 HU). As the boundaries for the articular cartilage was not clearly identifiable from the CT images, it was assumed that the joint interface between the femoral head and the acetabulum was covered with a uniform cartilage thickness of 1.0 mm [21]. The pelvis and proximal femur were meshed by tetrahedral and hexahedral elements consisted of 475,530 and 4920 elements (677,907 and 115,274 nodes), respectively. The cartilage was meshed by hexahedral elements, and number of nodes and elements were 2594 and 1220, respectively. An automatic calculation for global element edge length was used for mesh generation. To ensure numerical stability, linearly elastic hexahedral elements were used to mesh the cartilage layers (Figure 3). The material properties for the bone tissues and the cartilage were obtained from the literature (Table 1).

**Figure 4:**
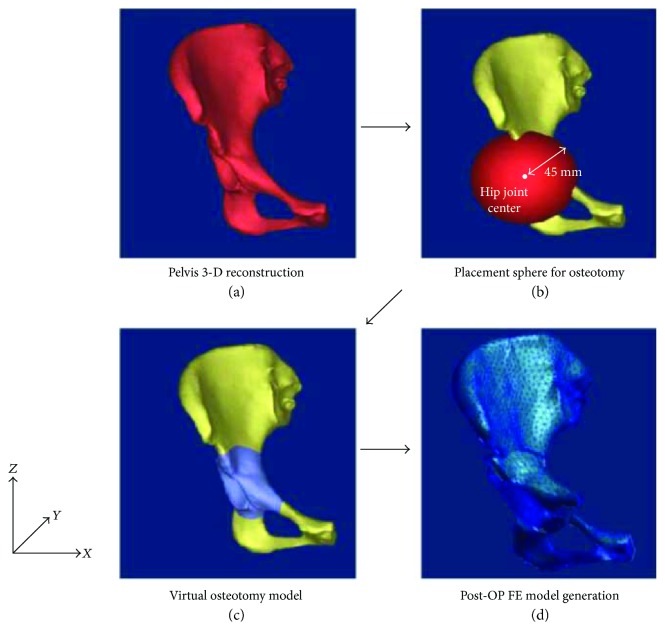
Procedure of the virtual PAO surgery: (a) 3-D reconstruction of the patient's pelvis, (b) superposition of the sphere (*r* = 45 mm) on the periphery of the acetabulum for osteotomy, (c) Boolean process to cut the pelvis and the acetabulum, and (d) 3-D rotation of the osteotomized acetabulum.

A simulated osteotomy was performed at the periphery of the acetabulum in the baseline model to mimic actual surgical procedure (Figure 4) [22]. Virtual cutting was done to simulate osteotomy due to PAO using Mimics software with a sphere (radius of 45 mm) located around the right hip center to separate the ilium, the ischium, and the pubis from the pelvis. The position of the central point of the sphere was matched with the central point made by geometry of the acetabular rim. The radius of the sphere was determined to include the whole regions of osteotomized acetabulum based on overlapped patient's CT images before and after PAO.

Theoretically, the “osteotomized” acetabulum could be reoriented to any desirable angles around the hip joint center. In this study, Post-OP FE models were generated, such that the amounts of the acetabulum rotations were varied according to the calculated actual acetabular rotation (ACR) during the surgery. In addition, the range of rotation of the osteotomized acetabulum by the surgery with respect to each axis was calculated based on Pre- and Post-OP patient's CT images. The preoperative FE model was rotated by 1/3 ACR in each axis incrementally up to 4/3 ACR. A total of twelve models were constructed by simulating incremental increase in the amount of the acetabular rotation (at an increment of 1/3 ACR) in each anatomical plane from 1/3 to 4/3 ACR. In other words, when incremental increasing of the acetabular angle through a single axis, rotating through the other axes was held constant. All Post-OP models were prepared using FE preprocessing software Patran (Version 2010, MSC Corp., USA).

### 2.3. Loading and Boundary Conditions

A finite-sliding surface-to-surface contact condition was defined at the joint interface between the femoral head and acetabulum. Contact constraints were enforced at articular surfaces based on the penalty method (ABAQUS 6.13, Simulia, RI, USA). The friction coefficient, *μ*, was set to 0.02 for simulating low-friction physiological condition in the presence of the synovial fluid [23]. In Post-OP models, the reoriented acetabulum was reconnected to the pelvis through tied contact to suggest complete bony union after the surgery.

Load conditions corresponding to those arising from a single-leg stance were simulated. A distributed load of 1177 N (231% of the body weight of the patient, 52 kg) was imparted to the distal end of the femoral shaft, while the superior region of the ilium and the symphysis pubis was fixed in all directions. Such loading and boundary conditions were assumed with abductor muscles counterbalancing the body weight as suggested by Bergmann et al. [24]. FE analysis was performed using a general-purpose FE solver ABAQUS (Simulia). Changes in the anatomical angles (CE angle, acetabular abduction, and acetabular anteversion) and the joint contact area, rate of changes in contact area, the contact pressure (95th percentile value) in relation to the directions, and amount of the rotations were assessed. All results were compared to the Pre-OP condition to determine the consequences of varied acetabulum rotations in three dimensions. For clarity, the difference was marked positive, if it indicated an increase in contact area/pressure, and negative if it indicated a decrease.

### 2.4. Sensitivity Analysis

Sensitivity analysis was performed to investigate changes of Pre-OP FE model's contact predictions due to variability in cartilage material properties and loading conditions. The baseline Young's modulus of the cartilage was altered by ±1 SD while the Poisson's ratio was kept constant. Then, Young's modulus was kept constant and the Poisson's ratio was deceased to 0.42 [25] and increased to 0.49 (with an assumption of cartilage incompressibility). The contact pressure (95th percentile value), mean contact pressure, and contact area were predicted for three different loading conditions [21], consisting of single-leg stance, normal walking, and stair climbing. A total of 15 models were evaluated for the sensitivity analysis.

## 3. Results

### 3.1. Acetabular Rotations during PAO Surgery

The Euclidean distance between individual landmarks had a mean ± standard deviation of 0.59 ± 0.15 mm, which confirmed the high repeatability in virtual marker placement, and the ICC was found to be 1.0 (*p* < 0.05) which is extremely high reliability. Our results provide evidence of position reliability between the observers. The calculated actual acetabular rotation (ACR) based on Pre- and Post-OP patient's CT images confirmed that the osteotomized acetabular rotation occurred in three dimensions. The actual ACR during PAO for a given patient expressed in Bryant angles was 9.7° in sagittal plane, 18° in coronal plane, and 4.3° in transverse plane.

### 3.2. Changes in Acetabulum Anatomical Angles

Anatomical angles were measured as acetabulum rotates (Table 2). In the coronal plane (rotation along *y*-axis), CE angle was gradually increased due to incremental increase in acetabulum rotations (from 12.5° to 26.7°), while the acetabular abduction was decreased as similar trend of changes of CE angle (from 47.4° to 29.7°). These parameters were restored to be within the normal range from the 18° rotation in the coronal plane. An analysis of changes in the acetabular anteversion was found not to be significant.

### 3.3. Changes in Joint Contact Areas

**Figure 5:**
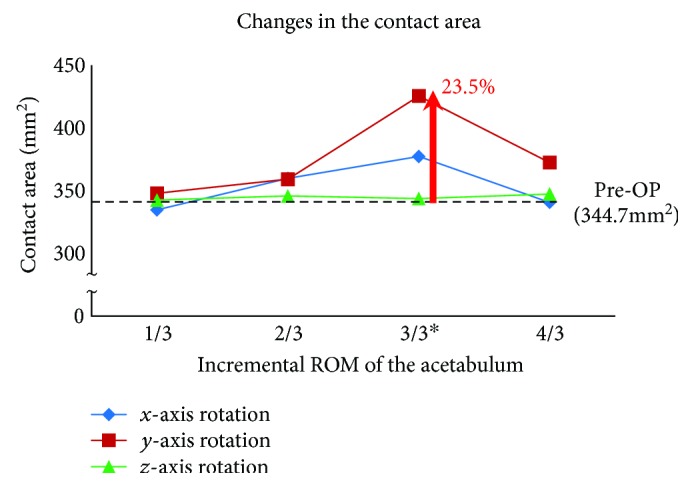
Changes in the contact area as a function of incremental increased rotations of the acetabulum as compared to the Pre-OP condition. The “^∗^” sign indicates the actual acetabular rotation (ACR) during the surgery.

**Figure 6:**
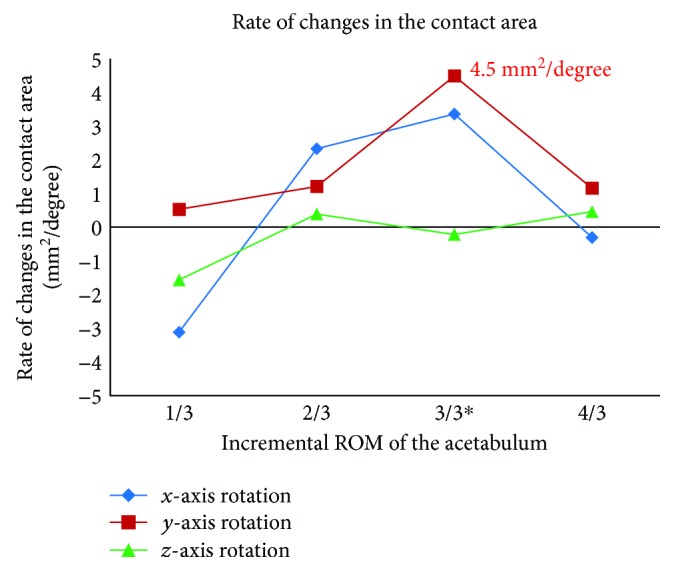
Rate of change in the contact area as a function of incremental increased rotations of the acetabulum. The negative sign indicates a decrease in contact area. The “^∗^” sign indicates the actual acetabular rotation (ACR) during the surgery.

Joint contact area due to incremental increase in acetabulum rotations showed the changes of −2.9%, 4.4%, 9.4%, and −1.2% in the sagittal plane (rotation along *x*-axis) and 0.9%, 4.2%, 23.5%, and 8.1% in the coronal plane (rotation along *y*-axis), respectively. In the transverse plane (rotation along *z*-axis), contact area and pressure remained relatively unchanged (differences less than ±1%). The above results were plotted in Figure 5. Maximum contact area was achieved for the sagittal plane and the coronal plane rotations. A most favourable increase of 23.5% in contact area (from 344.7 mm^2^ to 425.5 mm^2^) was seen for the 18° rotation in the coronal plane. An analysis of rate of changes in contact area showed the highest sensitivity of 4.5 mm^2^/degree for the coronal plane (Figure 6).

### 3.4. Changes in Contact Pressures (95th Percentile Value)

**Figure 7:**
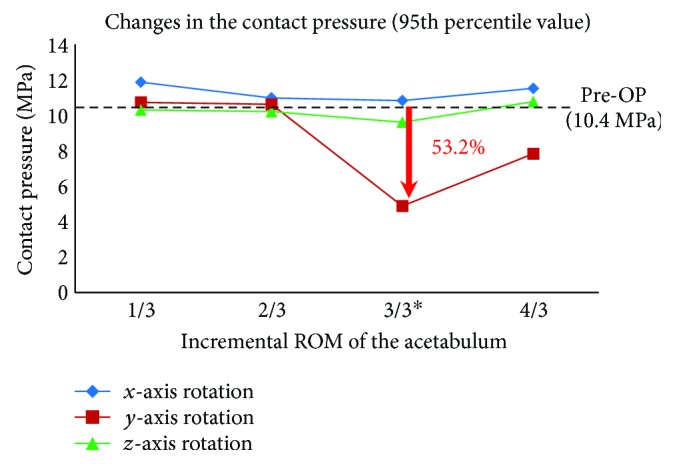
Changes in the contact pressure (95th percentile value) as a function of incremental increased rotations of the acetabulum as compared to the Pre-OP condition. The “^∗^” sign indicates the actual acetabular rotation (ACR) during the surgery.

**Figure 8:**
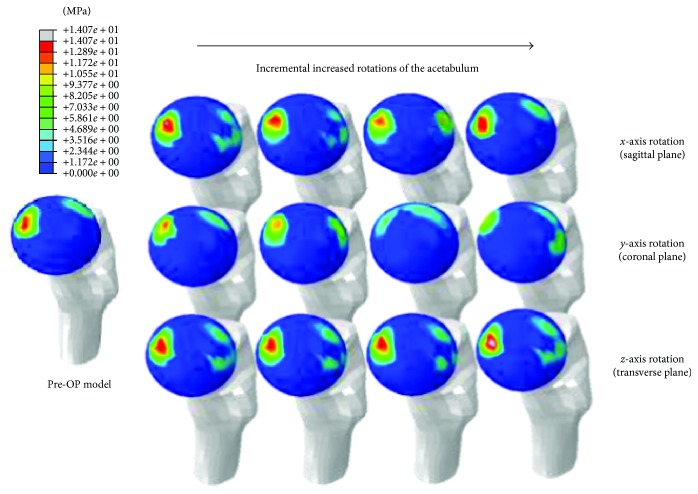
Contact pressure distributions of the Pre-OP model and twelve Post-OP models due to rotations of the osteotomized acetabulum in three anatomical planes.

There was clear trend in reduction in contact pressure as acetabulum rotates (Figure 7). Before PAO, contact pressure distributions showed stress concentrations on the superolateral regions of the femoral cartilage (Figure 8). Acetabular rotations resulted in increased contact area thus reduced pressure values. Contact pressure reached the lowest value in the model where the applied rotation of the acetabulum matched the actual acetabular rotation (ACR) during PAO. Similarly, the maximum pressure reduction was 53.2% (from 10.4 MPa to 4.9 MPa) found for the 18° acetabulum rotations in the coronal plane. A reduction of 19.2% in pressure was observed during acetabulum rotations in the transverse plane. The rate of change in contact pressure (95th percentile value) showed the highest sensitivity (0.3 MPa/degree) for acetabular rotations in the coronal plane.

### 3.5. Sensitivity Analysis

**Figure 9:**
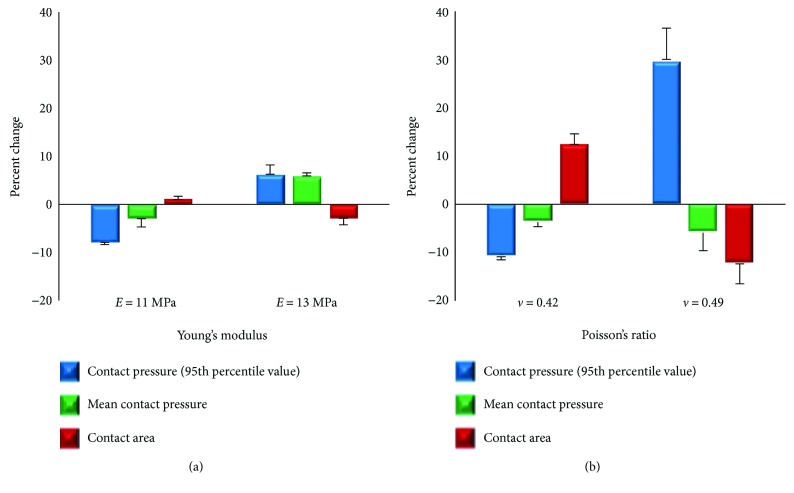
Percent changes in contact pressure (95th percentile value), mean contact pressure and contact area due to alterations in cartilage material properties, Young's modulus (a) and Poisson's ratio (b). Error bars indicate standard deviations over the three loading conditions evaluated.

Alterations of Young's modulus of cartilage resulted in approximately changes of contact pressure and mean contact pressure by ±7.1% and ±4.5%, respectively, and changes in contact area were about ±2% (Figure 9). When the Poisson's ratio was altered, contact pressure varied from −10.9 to 30.1%, and changes in contact area were approximately ±12.6%, while changes in mean contact pressure were less than 6%. Average RMS differences as compared to the baseline model were only about 3%.

## 4. Discussion

Computational simulations using patient's anatomy-specific models offer an attractive approach for prediction of key biomechanical parameters, such as hip joint contact patterns, before the PAO surgery. Currently, the clinical outcomes of the PAO remain controversial mainly because the procedure involves highly complex multiaxial rotations of the acetabulum while the optimal rotational parameters were unclear. While the osteotomy and rotation take place in three dimensions, but its pre- or intra-OP rotation depends on the experience and decision of the surgeon. In particular limited information on clinical and biomechanical efficacies in relation to amount and directions for rotation of the osteotomized acetabulum was reported. Therefore, we aimed to find out the most dominant factor regarding direction of the rotation due to incremental rotation of the osteotomized acetabulum in each axis. The effectiveness according to the three-dimensional rotations of the acetabulum was determined in terms of the contact stresses and the coverage area of the hip. The results obtained at different angles (joint realignments) provide quantitative information related to acetabular rotations to achieve the optimal outcome of the PAO surgical procedure.

In this study, virtual anatomic landmarks were created in the Pre- and Post-OP models in order to apply Bryant angles for calculating kinematic changes of the acetabulum. We verified the procedure of the landmark placement to ensure the repeatability of our method. Three landmarks of the pelvis were chosen, as suggested by Lycett and von Cramon-Taubadel [18], due to their clearly identifiable morphological features in CT images. Similar to the Lycett's approach [18], we quantified errors associated with anatomical landmark placement by applying Euclidean distance equation based on coordinates of the landmarks manually picked by six independent observers. The repeatability on anatomic landmarks set could be confirmed since Euclidean distance of less than 1.0 mm was calculated for individual landmarks.

Various studies on optimization of PAO have been reported [10–17]. Most studies have investigated the biomechanical effectiveness of PAO in relation to acetabular rotation. More specifically, the 3-D FE simulation-based optimal reorientation planning method was introduced by Liu et al. [11]. The study was performed subject-specific FE simulation for 4 subjects who underwent PAO surgery and evaluated biomechanical effect of the reorientation planning. However, the direction and amount of the acetabular rotation and the most dominantly affecting direction of the acetabular rotation during the actual PAO surgery have not been investigated. Therefore, using a kinematic analysis method, we determined the Bryant angle of the osteotomized acetabular rotation during PAO. Our results indicated that Bryant angles were 9.7° in the sagittal plane, 18° in the coronal plane, and 4.3° in the transverse plane. The results suggested that acetabulum rotated in three dimensions during the surgery. While the major component of acetabular rotations was in the coronal plane, rotations in other anatomical planes were also significant.

In our investigation, anatomical angles were assessed due to incremental adjustment of the acetabular rotations. CE angles were increased due to incremental increase in acetabulum rotations in the coronal plane and restored to be within the normal range (CE angle more than 20°) from adduction of 18° rotation [26]. In the same rotation, acetabular abduction also restored of normal range (35° ≤ acetabular abduction ≤ 45°) [27]. The acetabular anteversion of the dysplastic hip was reported because it was not significantly different from that of normal hips. In addition, the weight-bearing surface of the acetabulum is almost perpendicular to a vertical line in the standing position. Therefore, anatomical planes perpendicular to it are optimal for direct visualization and measurement of the surface and acetabular coverage; in practices, these are the coronal and sagittal planes [26]. The finding of changes in anatomical angles was compared with studies of long-term clinical outcome of PAO to improve clinical evidence of our study. Relatively few long-term outcome studies of PAO are available [2, 28–31]. In two long-term clinical studies (>10 years), the reported CE angles after PAO were 29.6 ± 6° (from 21° to 48°) [28] and 36.4 ± 6.5° (from 21° to 50°) [29], respectively. The reported acetabular abduction was 39.6 ± 3.9° (from 31° to 48°) [29]. In our FE analysis, the optimal CE angle (rotating from the initial adduction at 18°) and acetabular abduction angle (from the initial acetabulum rotations in the coronal plane at 18°) were 20.9° and 36.2°, respectively, which were comparable with the measured clinical outcomes. While it appears that the CE angle has fallen slightly out of the lower bound of the reported angular range, this could be attributed to the fact that only acetabular rotations were simulated in the study. If realistic translations of osteotomized acetabular during actual PAO surgery were applied, the accuracy of our biomechanical simulation results may be improved. However, such analysis involves surgical simulations of the acetabular motions in six degrees of freedom, which would require significant more efforts thus time for model preprocessing [8].

From FE analysis, we showed that adduction of 18° (the ACR in the coronal plane along *y*-axis) resulted in a most significant increase in contact area by 23.5% (from 344.7 to 425.5 mm^2^) compared to Pre-OP condition. Due to increased contact area, the corresponding contact pressure (95th percentile value) was reduced by 53.2% (from 10.4 to 4.9 MPa). Especially, peak contact pressure in Pre-OP model was concentrated on the superolateral regions of cartilage. However, contact pressures were reduced and evenly distributed around the superior regions of the cartilage due to incremental adjustment of the acetabular rotation in the coronal plane. While contact distributions of rotation in the sagittal and transverse planes were relatively unchanged (Figure 8), these changes improved contact area and peak contact pressure to the level close to the normal range [32–34]. Sensitivity of contact area and peak contact pressure according to the rotation direction of the acetabulum also showed the highest value (4.5 mm^2^/degree and 0.3 MPa/degree) at adduction of 18°. Thus, the coronal plane (adduction) turned out to be the most important rotation plane that strongly affects hip contact mechanics as compared to other planes (sagittal plane, *x*-axis; transverse plane, *z*-axis) for a given patient. In clinical scenarios, the actual amount of acetabulum rotations during PAO is mainly determined by surgeons. Our results seem to suggest that, to ensure clinical benefits of the procedure, acetabulum rotations in the coronal plane are of critical importance during the PAO.

The FE models used in this study were constructed based on CT images of a patient who underwent the PAO surgery. FE analysis requires robust validation process. We carefully compared our model results against Anderson's cadaveric experimental study on a normal hip joint [21] and Russell's FE analysis on a dysplastic hip model [25]. The contact area predicted by our Post-OP FE model was 428.8 mm^2^ and peak contact pressure 4.6 MPa, which agreed with the average contact area (321.9–425.1 mm^2^) and contact pressure (4.4–5.0 MPa) measured in Anderson's experimental study [21]. Due to the poor acetabular coverage, the contact pressure (95th percentile value) predicted by our Pre-OP dysplastic hip model was 10.4 MPa, which were higher than those measured in normal hips. Unfortunately, the experimental data on joint contact pressures at the dysplastic hip model is still significantly lacking. Nevertheless, Russell et al. conducted the FE analysis on a dysplastic hip model and the predicted peak contact pressure was 9.9 MPa [25]. However, due to the variations in acetabular coverage between different patients, direct comparison between the models may be difficult. The model predictions, in terms of joint contact pressure and contact area, compared favourably with those previous studies. In addition, since our study focused on evaluating relative performance of the same model and only relative changes were made for different hip alignment angles, the simulation results presented in this study should be thus considered clinically meaningful.

The cartilage Young's modulus alternations did not significantly affect the FE results of contact pressure (95th percentile value), mean contact pressure, and contact area (±7.1%, ±4.5%, and ±2%, resp.) from the baseline case. When Poisson's ratio decreased from 0.45 to 0.42, the peak pressure was decreased by 10.9% due to an increased contact area by about 12.4%. However, as the Poisson's ratio increased to nearly incompressible materials (*ν* = 0.49), contact pressure was remarkably increased by approximately 30.1%; however, the mean contact pressure was only changed by 6%. These results suggested that the proper choice for Poisson's ratio is more critical for accurate prediction of peak contact pressure, as compared to the mean contact pressure. In addition, a decrease in mean contact pressure was considered to be indicative of a general decrease in joint contact stresses, which is aligned with clinical goal of the PAO surgery. Similar approaches have been adopted in FE analysis performed for the understanding of the hip and elbow joint contact mechanics [21, 35].

A number of modelling assumptions were also made to facilitate FE analysis in the study. Firstly, linear-elastic, homogeneous material properties were used to model bone and cartilage tissues. In most FE simulation studies of PAO [12, 15, 16], the bony structure and cartilage were modeled as the linear-elastic, homogeneous materials. It was reported that the effects of using linear-elastic, homogeneous material properties on predicted cartilage stresses were negligible [36]. While the cartilage is reported as a biphasic material with time-dependent mechanical behavior, the frequency for walking loads is in the order of 1 Hz. Thus, time-dependent response of the cartilage can be neglected [37]. Secondly, the cartilages were not clearly identifiable from the CT images (using the clinical CT scanner), and a constant thickness of 1.0 mm was assumed for articulating surfaces of the acetabulum and the femoral head. This enabled us to evaluate contact mechanisms on the cartilage in DDH patients similar to a previous study [32]. Modeling subject's anatomy-specific cartilage layer seems to be essential for our FE analysis. However, it has been reported that the predicted optimal alignment of the acetabulum was not significantly sensitive to the change of the cartilage thickness distribution during PAO [14]. Thirdly, an osteotomy gap between the osteotomized acetabulum and the pelvis was assumed a perfect fusion after PAO [12]. Clinically, the osteotomy gap is initially fixed with biodegradable fixation screws and then fused over a period of time [15]. Thus, our Post-OP FE models did not include the potential effects of model instability due to osteotomy gap and insufficient screw fixation after the surgery. The effects of such model instability on joint contact interactions remain to be investigated. Fourthly, it was acknowledged that CT scan was performed in the supine position and the FE models simulated a single-leg stance scenario [24]. Niknafs et al. [14] found no significant difference between the contact pressure in the single-leg stance reference frame and those in the supine reference frame. Lastly, we performed anatomical-specific FE analysis for only one patient. For providing clinical evidence, our finding of measured anatomical angles from each Post-OP model was compared with long-term clinical outcomes of several studies. Overall, our results on the measured anatomical angles accord with the restored range as measured in long-term clinical studies. Therefore, the methodology developed in this study is equally applicable in studies involving more patients. The loads based on in vivo measurements were applied, but the acetabular geometry and pattern of loading may vary for each subject. To increase the accuracy of simulation, a large-scale analysis is thought to be necessary for guideline provision for surgical planning depending on the shape of the diseased femoral head and the acetabulum, as well as the severity of DDH in patients.

## 5. Conclusions

The results of our study show that the acetabulum rotation in the coronal plane (adduction) had the strongest effects on contact pressure and contact pressure compared to rotations in other planes. In particular, the osteotomized acetabulum rotation with adduction of 18° is considered to be the most effective angle for the given patient. Although this study was limited to a single patient, the methodology developed in this study could contribute to the preoperative planning that determines the optimal direction and amount of rotation of the osteotomized acetabulum in three dimensions during PAO.

## Figures and Tables

**Table 1:** Material properties used in the hip FE models.

Components	Young's modulus (MPa)	Poisson's ratio	Ref.
Cortical bone	17,000	0.3	[19]
Cancellous bone	100	0.2	
Cartilage	12	0.45	[20]

**Table 2:** Changes in anatomical angles of Post-OP models due to incremental increase in acetabulum rotations. The “^∗^” sign indicated to be within the normal range.

Rotation axis	Incremental ROM of acetabulum	CE angle	Acetabular abduction	Acetabular anteversion
Pre-OP model		12.2°	48.1°	10.8°

Post-OP models				

Sagittal plane (*x*-axis)	1/3	9.7°	51°	13.5°
	2/3	10.5°	51.3°	14.6°
	3/3	11.7°	49.4°	15.6°
	4/3	10.9°	48.5°	16.2°

Coronal plane (*y*-axis)	1/3	12.5°	47.4°	15°
	2/3	15.9°	41.9°	10.5°
	3/3	^∗^ **20.9°**	^∗^ **36.2°**	10.7°
	4/3	^∗^ **26.7°**	29.7°	8.5°

Transverse plane (*z*-axis)	1/3	9°	50.4°	12.1°
	2/3	9.8°	50.6°	11.5°
	3/3	10°	51.4°	11.5°
	4/3	11.9°	49.5°	13.5°`
